# Characterization of Oral Microbiota in Cats: Novel Insights on the Potential Role of Fungi in Feline Chronic Gingivostomatitis

**DOI:** 10.3390/pathogens10070904

**Published:** 2021-07-17

**Authors:** Janina A. Krumbeck, Alexander M. Reiter, James C. Pohl, Shuiquan Tang, Young J. Kim, Annika Linde, Aishani Prem, Tonatiuh Melgarejo

**Affiliations:** 1 MiDOG LLC, Irvine, CA 92614, USA; jkrumbeck@zymoresearch.com (J.A.K.); stang@zymoresearch.com (S.T.); aprem@zymoresearch.com (A.P.); 2 School of Veterinary Medicine, University of Pennsylvania, Philadelphia, PA 19104, USA; reiter@vet.upenn.edu; 3 College of Veterinary Medicine, Western University of Health Sciences, Pomona, CA 91766, USA; james.pohl@westernu.edu (J.C.P.); yjkim@westernu.edu (Y.J.K.); alinde@westernu.edu (A.L.)

**Keywords:** next-generation DNA sequencing, oral microbiota, microbiome, mycobiota, 16S rRNA, fungal ITS, feline stomatitis

## Abstract

Previous studies have suggested the involvement of viral and bacterial components in the initiation and progression of feline chronic gingivostomatitis (FCGS), but the role of fungi remains entirely unknown. This pilot study aimed to investigate the bacteriome and mycobiome in feline oral health and disease. Physical exams, including oral health assessment, of privately owned, clinically healthy (CH) cats (*n* = 14) and cats affected by FCGS (*n* = 14) were performed. Using a sterile swab, oral tissue surfaces of CH and FCGS cats were sampled and submitted for 16S rRNA and ITS-2 next-generation DNA sequencing. A high number of fungal species (*n* = 186) was detected, with *Malassezia restricta*, *Malassezia arunalokei*, *Cladosporium penidielloides/salinae*, and *Aspergillaceae* sp. being significantly enriched in FCGS samples, and *Saccharomyces cerevisiae* in CH samples. The bacteriome was significantly distinct between groups, and significant inter-kingdom interactions were documented. *Bergeyella zoohelcum* was identified as a potential biomarker of a healthy feline oral microbiome. These data suggest that fungi might play a role in the etiology and pathogenesis of FCGS, and that oral health should not simply be regarded as the absence of microbial infections. Instead, it may be viewed as the biological interactions between bacterial and fungal populations that coexist to preserve a complex equilibrium in the microenvironment of the mouth. Additional investigations are needed to improve our understanding of the feline oral ecosystem and the potential interactions between viruses, bacteria, and fungi in FCGS.

## 1. Introduction

The prevalence of feline chronic gingivostomatitis (FCGS) is approximately 0.7–12% [1]. Affected cats usually present with oral inflammation, pain, anorexia and dysphagia. Feline calicivirus (FCV) and feline herpesvirus-1 (FHV-1) have been implicated in the development of the disease, but it has also been hypothesized that cats with FCGS may show an aberrant reaction to plaque bacteria that leads to oral inflammation beyond gingivitis and periodontitis [2]. However, the exact cause of FCGS remains unknown, and there is no gold standard in terms of treatment besides partial and full-mouth tooth extraction and extensive medical management. Consequently, FCGS can be a challenging disease, not only for clinicians but also for clients [3]. Current therapeutic approaches include professional dental cleaning, tooth extractions (partial or full mouth), and extensive medical (antimicrobial, anti-inflammatory, immunosuppressive, immunomodulatory) management. Antibiotic therapy is included in most management plans, but bacterial culture and sensitivity testing are not typically performed. Empirical treatment of FCGS with amoxicillin, amoxicillin clavulanate, clindamycin or metronidazole without proper diagnostic information can promote bacterial resistance and thus become ineffective.

To better understand the biological role of the microbial populations on the development of FCGS, two scientific groups partially described the cat oral microbiome using culture-based methods [4,5]. These studies showed that the feline oral cavity is typically polymicrobial with a preponderance of obligate anaerobic and facultative anaerobic bacteria, of which only a few bacterial strains are cultivatable using routine laboratory methods [6]. Molecular microbial tools, like next-generation DNA sequencing (NGS), have the advantage that they are culture-independent and more sensitive. NGS uses an untargeted sequencing approach and can identify and quantify bacteria and fungi present in a sample, including previously unknown microbes. This technique has been previously applied to study the feline oral cavity in health [7,8,9,10,11] and disease [12,13,14,15,16,17], showing that the bacterial profile in the mouth of cats is rich and highly diverse. In clinically healthy cats, *Porphyromonas, Moraxella*, and *Fusobacterium* are the most abundant genera [18], and *Capnocytophaga canimorsus* is the most prevalent species, followed by *Xanthomonadaceae* and *Bergeyella* spp. [10]. In cats affected by gingivitis, mild periodontitis, or other oral diseases, the key pathogens from the phyla *Spirochaetes* and *Bacteroidetes* [11] and the *Peptostreptococcaceae* family [13] were dominant. Another study showed that members of *Pasteurella multocida* subsp. *multocida* were significantly more prevalent in diseased cats compared to controls [5].

Besides bacteria, fungi are an important part of the mammal microbiome in various species [19] and infections [16,20]. To date, only two studies have investigated fungal oral microbiota, i.e., mycobiota, of healthy [9] or allergic cats [21]. To our knowledge, there is no study investigating the oral mycobiome of cats with FCGS. Understanding the role of oral microbiota in FCGS could provide critical insight into disease pathophysiology as well as outline new approaches for prevention and treatment. The aim of the present study was to investigate the bacterial and fungal microbiota of CH cats and cats with FCGS. It was hypothesized that the feline oral microbial composition and structure will differ between the two groups and that these differences and population dynamics may provide clinicians with a better understanding of the complex etiology of and potential therapeutic approaches to FCGS.

## 2. Results

**Figure 1:**
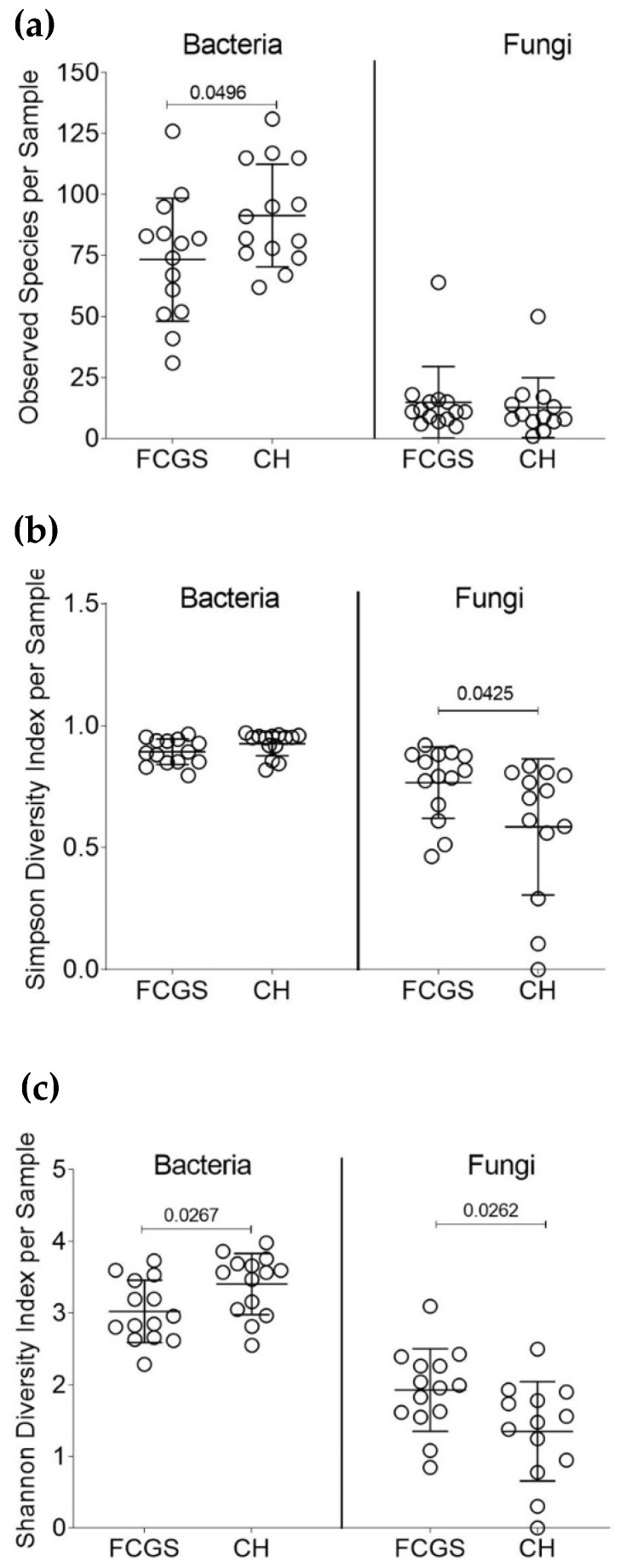
Microbial diversity analysis of FCGS and CH samples. Significant *p*-values are shown in the graphs. (**a**) Number of observed species for bacteria and fungi for each group. (**b**) Shannon Diversity Index for each sample. (**c**) Simpson Diversity Index for each sample.

**Figure 2:**
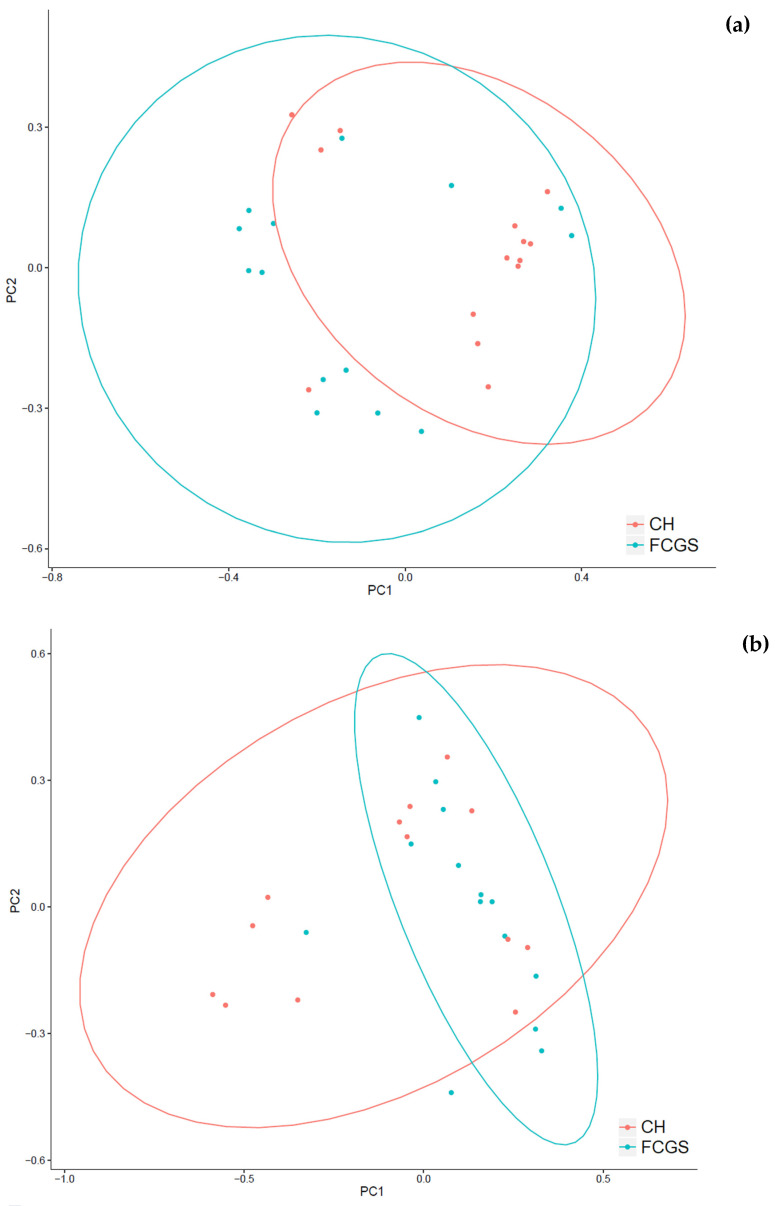
Beta diversity (Bray–Curtis-distance) for microbial composition of (**a**) bacteria and (**b**) fungi. Shown are CH samples in red and FCGS samples in blue (*p*-value > 0.05).

A total of 249 bacterial and 186 fungal genera were identified in this dataset. All samples harbored both bacteria and fungi, with only one exception of a CH sample (CH_5) that apparently had no fungal species present. A commonly used measurement tool for the microbial diversity within samples, i.e., how many different types of microbes are present in a sample, is the alpha-diversity (Figure 1a–c). The alpha-diversity measurements for CH and FCGS samples showed that the number of observed bacterial species was significantly higher than the number of fungal species. There was a mean of 76 and 90 (rounded) bacterial species in the FCGS and CH samples, respectively. However, no significant differences were found with regards to the number of fungal species between FCGS and CH samples with a mean of 15 and 13 (rounded), respectively (Figure 1a). The bacterial diversity was significantly different between the groups for both the number of observed species and the Shannon diversity index (a value that increases with biodiversity), showing a higher alpha-diversity in the CH samples (Figure 1a,c). For the fungal profile, both the Shannon diversity index and the Simpson diversity index—which measures the relative abundance of species—were significantly higher in FCGS samples compared to CH samples (Figure 1b,c). The beta-diversity—a measurement of microbial diversity between samples—showed that FCGS samples were not significantly distinct from the CH samples for the bacterial or fungal composition (Figure 2a,b).

### 2.1. Analysis of the Bacterial Microbiota in CH and FCGS Cats

**Figure 3:**
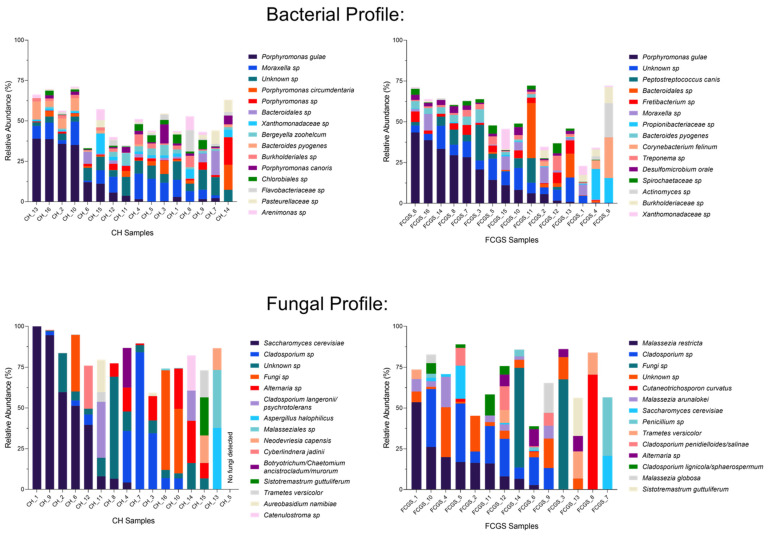
Microbial taxa summary plots for bacteria (top panels) and fungi (bottom panels) in oral samples. The left-side of the figures shows CH samples, the right-side FCGS samples. Each column represents the microbial profile of one cat. Shown are the top 15 most abundant species per group, with the most abundant species being listed first.

The bacterial composition profile was analyzed in each group (Figure 3, top). In healthy cats, the top five most dominant bacterial species on average were *Porphyromonas gulae* (10.0%), *Moraxella* sp. (7.3%), *Porphyromonas* sp. (2.8%) *Porphyromonas circumdentaria* (2.6%), and *Bacteroidales* sp. (2.6%). The top five most dominant bacterial species based on average relative abundances in FCGS samples were *Porphyromonas gulae* (16.9%), *Peptostreptococcus canis* (4.9%), *Bacteroidales* sp. (3.8%), *Fretibacterium* sp. (3.5%), and *Propionibacteriaceae* sp. (2.7%). No bacterial species represented more than 50% of the microbiota in any sample. A commonly known member of the normal oral microbiota in cats, the genus *Moraxella*, was present at a 5.8% average abundance.

**Figure 4:**
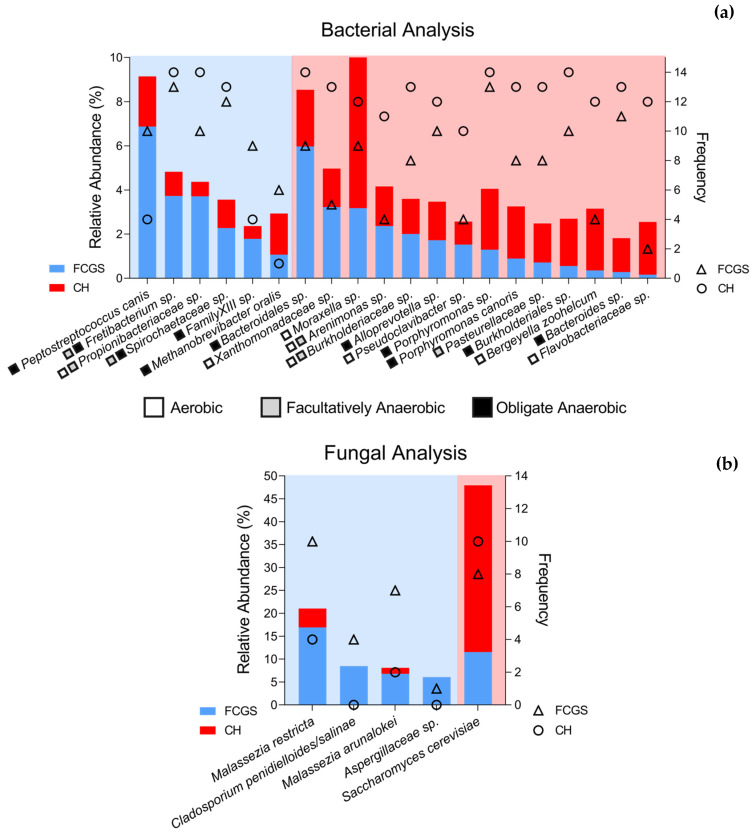
LEfSe analysis summary for the (**a**) bacterial and (**b**) fungal species level highlighting species that are significantly different between groups. Bar graphs show the relative abundance of a given species in those cats that had that taxa present (left y-axis). Triangles and circles show the frequency of that given species in the dataset per group; i.e., how many cats had that species present in their microbiota (right y-axis). Shown in blue and triangles are FCGS samples, shown in red and circles are CH samples. The oxygen dependency of bacterial species is indicated by a white box for aerobic bacteria, a grey box for facultatively anaerobic, and a black box for anaerobic bacteria.

A LEfSe (Linear discriminant analysis Effect Size) analysis was performed to identify the constituents of the bacterial microbiota that differed between groups at all taxonomic levels. Five families were significantly enriched in FCGS samples (highlighted in green) as shown in Supplemental Figure S1a, and 14 in CH samples (highlighted in red). At the species level, 16 bacterial species were significantly more abundant in FCGS samples and 39 bacterial species in CH samples. After filtering those species that had at least 1.5% relative abundance in one of the groups, six species remained in the FCGS group and 14 in the CH group (Figure 4a). Of the 20 bacteria identified in both groups, nine were obligate anaerobes. While the CH group had a significantly higher number of aerobic bacteria, such as *Xanthomonadaceae* sp., *Moraxella* sp., *Pseudoclavibacter* sp., *Bergeyella zoohelcum*, *Flavobacterium* sp., and *Flavobacteriaceae* sp., the species enriched in the FCGS group were all either obligate or facultative anaerobic with only one exception of *Propionibacteriaceae* sp., which is either aerobic or facultative anaerobic. Interestingly, *Fretibacterium* sp., *Propionibacteriaceae* sp., *Spirochaetaceae* sp., and *Lachnospiraceae* sp. had higher frequencies in CH samples (i.e., they were found more often in this sample set), but their relative abundances were significantly higher in FCGS samples. There was no species that was significantly more abundant in CH samples but was seen more frequently in FCGS samples than in CH samples.

### 2.2. Analysis of the Fungal Microbiota (Mycobiota) in CH and FCGS Cats

Of the 186 different fungal species (or 123 genera) that were identified across the dataset, the top five most dominant fungal species based on average relative abundances in CH cats were *Saccharomyces cerevisiae* (28.0%), *Cladosporium* sp. (13.0%), *Alternaria* sp. (6.9%), *Fungi* sp. (5.7%), and *Cladosporium langeronii/psychrotolerans* (4.1%). Six samples (CH_1, CH_9, CH_2, CH_6, CH_7, and CH_16, ordered as seen in Figure 3) were dominated by a single fungal organism (*Saccharomyces cerevisiae* (4), *Cladosporium* sp. (1), and *Fungi* sp. (1), respectively), representing more than 50% of the mycobiota in those samples (Figure 3, bottom). In FCGS samples, the top five most dominant fungal species (average) were *Malassezia restricta* (11.9%), *Cladosporium* sp. (11.5%), *Fungi* sp. (9.2%), *Saccharomyces cerevisiae* (5.8%), and *Cutaneotrichosporon curvatus* (5.2%). The fungal profile is characterized by a large degree of variation between samples. Four samples (FCGS_1, FCGS_14, FCGS_3 and FCGS_8, ordered as seen in Figure 3) were dominated by a single fungal organism (*Fungi* sp. (2), *Malassezia restricta* (1), and *Cutaneotrichosporon curvatus* (1), respectively), representing more than 50% of the mycobiota in those samples.

To identify members of the mycobiota that could differentiate the two groups, an LDA Effect Size (LEfSe) analysis was conducted. This test identifies statistically significant fungal taxa at all taxonomic levels (phylum to species) that characterize the differences between the CH and FCGS cat mycobiota. The LEfSe analysis showed that two classes (*Eurotiomycetes* and *Malasseziomycetes*), two orders (*Eurotiales* and *Malasseziales*), and two families (*Aspergillaceae* and *Malasseziaceae*) were significantly enriched in FCGS samples (Supplementary Figure S1b). In CH samples, only one family (*Saccharomycetaceae*) was significantly enriched. At the species level, four fungal species were significantly more abundant and frequent in FCGS samples, specifically *M. restricta*, *Cladosporium penidielliodes-salinae*, *M. arunalokei*, and *Aspergillaceae* sp. Only one species, *Saccharomyces cerevisiae*, was significantly enriched in CH cats (Figure 4b, and Supplementary Table S1). For this analysis, the average relative abundance was calculated for only those samples that had the species present, as opposed to the full dataset as shown above. With the exception of *Cladosporium penidielliodes-salinae* and *Aspergillaceae* sp., all fungi were found in both FCGS and CH samples.

### 2.3. Microbiota Core Analysis

**Figure 5:**
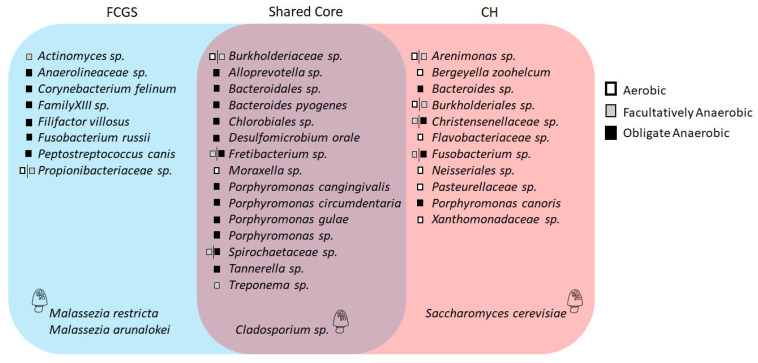
Microbial core analysis. This graph shows those bacterial and fungal species that are part of the FCGS core, CH core, or shared feline oral core microbiota. Fungi are indicated by a cartoon-fungus. The oxygen dependency of bacterial species is indicated by a white box for aerobic bacteria, a grey box for facultatively anaerobic, and a black box for anaerobic bacteria.

Core analysis was conducted to identify core members of the microbiota that are (1) shared between the two groups (i.e., which ones are part of the feline oral cavity independently of the health status), and which ones are part of the core for (2) the CH group, or (3) the FCGS group. For a species to be considered part of the core microbiota, the species had to be present in at least 50% of the samples and represent at least 1% of the microbiota in that sample set. This analysis confirmed the findings of the LEfSe analysis of the mycobiota and showed that *Cladosporium* sp. was the only fungal species shared between the groups, while *M. restricta* and *M. arunalokei* were part of the FCGS core, and *S. cerevisiae* part of the CH core microbiota only (Figure 5). Even though *C. penidielloides/salinae* was significantly enriched in the FCGS samples, it was not part of the FCGS core mycobiota.

The bacterial core microbiota analysis showed that 15 species were part of the shared core, several of which were previously identified in the LEfSe analysis (Figure 4a). There were only two species identified in the CH samples that were obligate anaerobic, all other species were either aerobic (5) or facultative anaerobic (4). Interestingly, *B. zoohelcum* and *P. canoris* were also part of the CH core (Figure 5).

### 2.4. Co-occurrence Analysis

**Figure 6:**
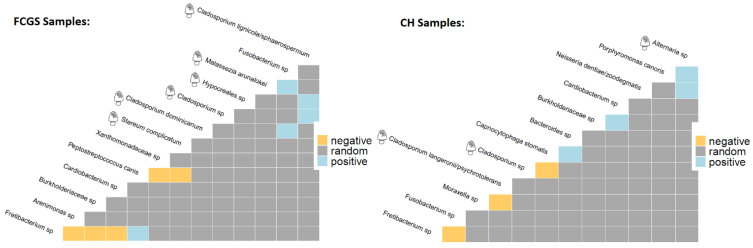
Heatmap of co-occurrence analysis for FCGS (**left**) and CH (**right**) samples. This analysis combined both bacteria and fungi. Negative co-occurrences are marked in yellow, positive occurrences in blue, and random occurrences in grey. Fungi are indicated by a cartoon-fungus for faster identification.

To better understand the population dynamics of this ecosystem, a combined co-occurrence analysis for both bacteria and fungi was conducted (Figure 6). This analysis showed that in FCGS samples *Fretibacterium* sp., a species significantly enriched in the FCGS group (Figure 4a), exhibited negative co-occurrences with *Arenimonas* sp. and *Burkholderiaceae* sp., two species that are significantly enriched in the CH group and part of the CH core microbiota (Figure 5). Similarly, *P. canis*, which is significantly enriched in the FCGS group and a core member, exhibited a negative co-occurrence with *Xanthomonadaceae* sp., which is significantly enriched in the CH group and a CH core member. Furthermore, in the FCGS group, four fungi were detected to have positive co-occurrences among the same kingdom. This phenomenon was not seen in the CH group. The CH group had only one co-occurrence between two bacterial species that were both previously identified in the analysis. More specifically, a negative co-occurrence between *Fusobacterium* sp., a CH core member (Figure 5), and *Fretibacterium* sp., a shared core member that was significantly enriched in the FCGS group (Figure 4a, and Figure 5).

## 3. Discussion

Our pilot study showed that the feline oral microbiota is distinct between CH and FCGS samples with respect to bacterial and fungal composition. Both groups were characterized by a high degree of microbial diversity for both bacteria and fungi (Figure 2). Specifically, the FCGS samples showed a higher alpha-diversity than healthy samples (Figure 1). A large degree of sample heterogeneity within the groups was seen, showing that a subset of CH samples had a mycobiota profile that was dominated by a single fungus (*n* = 6).

According to the LEfSe analysis, which identifies species that best characterize each group, several taxa were clearly distinct between the groups (Figure 4), including commonly known pathogens such as *M. restricta* and *P. canis*, as well as known commensals (i.e., *Pasteurellaceae* and *Moraxella*). These commensals were significantly more abundant in CH samples. Furthermore, we demonstrated that four fungal species were significantly enriched in FCGS samples and *S. cerevisiae* in CH samples (Figure 4b). To further understand the population dynamics of the microbiota a co-occurrence analysis was conducted. This analysis identified several significant correlations between bacterial and fungal taxa (Figure 5), and showed that numerous bacterial species seemingly coexist with a smaller number of fungal species.

The use of NGS technology as a tool to investigate the oral microbiota composition of cats suffering from FCGS has the advantage of capturing the total microbiota composition as opposed to culture-dependent methods that report on only those microbes that can grow under given laboratory conditions. The NGS technique described here offers detailed insight into microbial population-wide dynamics, showing how they are distinct between CH and FCGS cats. Data presented may help clinicians understand the complexity of microbiota involved in oral health and disease [18]. This could serve as a basis for the development of novel future treatments.

Traditionally, a higher microbial diversity of a given body site is correlated to better health of patients [8,22]. The data obtained in the present study confirmed this concept of bacterial diversity and health (Figure 1a,c). However, a previously published study in cats suffering from FCGS demonstrated a higher microbial diversity in cats with FCGS [18], which is contrary to the results obtained here. Another study observed no differences in the diversity of the bacterial profile of cats with and without chronic gingivitis [23]. The mycological analysis, however, showed a significantly lower fungal diversity in healthy cats compared to cats with FCGS (Figure 1b,c). As no previously published study has investigated the mycobiota in CH and FCGS cats, this is a novel finding.

The overall composition of the bacteriome and mycobiota showed a large degree of variation between individuals in each group (Figure 3), and several previously reported bacterial commensals and oral pathogens were detected. To further dissect the microbial profiles per group, the comparative analyses of the two groups using LEfSe showed that CH cats have a clearly distinct bacterial and fungal microbiota compared to FCGS cats. Specifically, CH cats harbored a larger number of commensal organisms that have previously been reported, such as *Moraxella* [17] and *Pasteurellaceae* [4]. Three species stand out in the CH group, including *F. nucleatum*, *P. canoris*, and *B. zoohelcum*. 

*F. nucleatum* is known to be an indicator for gingivitis, but is also known as a “bridge” between early and late colonizers of dental plaque [24]. *P. canoris* has been previously reported in a low number (8%) of dogs with gingivitis [25] and in one cat suffering from FCGS [5]. In the present study, both species were identified in CH and FCGS groups, but the frequencies as well as the relative average abundances were higher in the CH group. Additionally, *P. canoris* was part of the CH core microbiota. This highlights the importance of further investigation of the potential role of the two species in FCGS. Finally, *B. zoohelcum*, a species associated with a healthy animal oral microbiota [26], was significantly enriched in the CH group and part of the CH core microbiota. It could therefore be used as a marker of a healthy feline oral microbiota, if larger studies support this finding. Overall, the CH microbiota was characterized by a higher number of oxygen-dependent species than the FCGS microbiota, which aligns with previous studies that have shown plaque formation is associated with a higher abundance of anaerobic bacteria [27]. *Neisseriaceae* species are associated with a healthy oral microbiota [10], and while this taxon was not significantly enriched in CH samples, *Neisseriales* was still a part of the CH core microbiota. 

Cats with FCGS harbor commonly known bacterial pathogens such as *Fretibacterium* sp. and *Peptostreptococcus*, which is consistent with previously published work [11]. Two species, *P. canis* and *M. oralis*, were especially interesting as they were significantly enriched, and *P. canis* was also part of the FCGS core. Both are anaerobic species that have previously been reported in periodontitis; *P. canis* is a late colonizer of canine periodontitis [28], and *M. oralis* is a relatively novel oral pathogen discovered in 2013 [29]. With the exception of *P. canis*, none of these species showed a significant co-occurrence with other members of the bacterial or fungal microbiota. *P. canis*, significantly enriched in FCGS samples and a FCGS core member, had a negative co-occurrence with *Xanthomonadaceae* sp., which is significantly enriched in CH samples and a CH core member. This may suggest an antagonistic relationship between the two taxa. A significant enrichment of *Xanthomonadaceae* was also previously reported for cats without gingivitis, which aligns with the data presented here [23]. In vitro studies may be needed to further investigate this.

For the first time, the feline mycobiota in FCGS samples were analyzed. This analysis showed that cats with FCGS harbor a commonly known human scalp pathogen, *Malassezia restricta* [30] at a significantly higher abundance and frequency than CH cats. *Malassezia arunalokei* is a relatively unknown fungus that was just discovered in 2016 [31] and has, so far, never been reported in association with FCGS. Studies in human patients have reported that *Malassezia* species could be considered commensals of the oral mycobiota [32]. Future studies are needed to understand the role of this fungus in feline health and disease, especially since a symbiotic relationship with the residential bacterial microbiota and the host is needed for fungi to survive and grow in the oral cavity [24]. Interestingly, a higher abundance of *Malassezia arunalokei* correlated negatively with the abundance of another fungus, *Cladosporium lignicola/spaerosperum*. As no further metagenomic or in vitro data are available to discuss the role of *M. arunalokei* in feline health, any conclusion would be speculative. *Cladosporium* species were significantly more abundant in FCGS samples, but little is known about the health implications of this genus in cats. *Cladosporium sphaerospermum* has previously been associated with the development of allergies and asthma symptoms in humans [33]. Different species of *Aspergillus* have been reported to cause various feline diseases [34,35,36]. In the present study, *Aspergillaceae* sp. was significantly enriched in FCGS samples, and even though the frequency was low with only one positive sample, the taxon was not detected in any of the CH samples. The same applies to the other fungal species detected, *Cladosporium penidielloides/salinae* (4 out of 14 FCGS samples were positive [28.6%]), which was not present in the CH samples. A larger study cohort may confirm these two species as potential biomarkers for FCGS in cats. Interestingly, only one fungal species, *Saccharomyces cerevisiae*, was enriched in CH samples and part of the CH core. Very limited data about any potential role of *S. cerevisiae* in feline health are available at this time. Different strains of *S. cerevisiae* have been used as a probiotic supplement in humans [37] and animals [38], and a potential beneficial effect of this species in feline health would be worthy of further exploration.

Fungi and bacteria form close inter-kingdom ecosystems in confined spaces such as the oral cavity. While fungi are outnumbered by the bacterial cell counts, the fungal biomass is considerably larger—up to 1000 times the size of bacterial cells [39]. Fungi can switch between a yeast and filamentous form, which has implications on their virulence, carbon and oxygen metabolism, size, and interactions with other members of the oral microbiota [40]. Specifically, fungi like *Candida albicans* are known to form pathogenic biofilms with different bacteria of the microbiota, including *S. mutans* [41]. Such biofilms are characterized by chemical gradients, cross-feeding between members, and antibiotic resistances. Potential biofilm formation or inter-species interaction between *Malassezia* or *Cladosporium*—the two fungal taxa of significant interest in the present study—and other bacteria have not been conducted in vitro or in vivo in the context of feline oral health or FCGS. Therefore, no clinical relevance about inter-species dependencies can be ascribed at this time. Fungi are proposed keystone species in the human oral cavity, an essential part of maintaining oral community stability and implicated in shifts towards the development of disease [24]. Since many oral diseases are associated with perturbations of community balance [24], further research is needed to understand how such a balance may look like in a healthy state to define oral health beyond the absence of disease [42]. A balance between the host and all intrinsic microorganisms is essential [24], especially since the majority of therapeutic methods are monotherapies, despite the fact that biofilms are polymicrobial.

A limitation of this study is its relatively low number of samples, descriptive nature, and that no transcriptomics data were obtained. To date, very little is known about the feline oral mycobiota [21], and essentially nothing about the mycobiota in the context of FCGS. This provides a challenge to put the results presented here into clinical context. This study suggests that the mycobiota may play a role in the development of FCGS. Consequently, a fungal culture, or better NGS, may be considered for future routine health assessments pending analytical validation of this assay. Future studies with larger sample sizes may focus on following FCGS cats from diagnosis of the disease throughout their treatment regimen to assess the microbial component during disease progression and/or remission. Finally, the timing of previous recent professional dental cleaning, tooth extraction, and extensive medical management was not recorded in the present study.

## 4. Materials and Methods

### 4.1. Subjects Included and Study Design

A total of 28 samples were analyzed in this study, 14 from clinically healthy (CH) cats and 14 from cats with FCGS. The CH samples were all collected at Western University of Health Sciences (WUHS), Pet Health Center (PHC), Pomona, CA (*n* = 14). The FCGS samples were obtained from WUHS-PHC (Pomona, CA, USA), Saddleback Animal Hospital (Tustin, CA, USA), Advanced Veterinary Specialty Group (Tustin, CA, USA), and Cat Care Clinic (Orange, CA, USA). Six different cat breeds were represented, across a wide age range (2 to 13 years old) with an average of 4 ± 0.5 years in the CH group and 8 ± 1.1 years in the FCGS group. A standardized upstream sampling protocol (see Section 4.2.) was used to ensure samples were collected and preserved on a consistent basis. The CH cats were recruited from the surgery section at the WUHS-PHC at the time of admission for elective procedures (e.g., neutering). All cats in the CH and FCGS groups underwent the same examination protocol and were classified based on a previously published FCGS scale with slight modifications [43]. Briefly, the oral cavity was examined thoroughly, and lesions were sorted according to severity, as follows: grade 0, absence of lesions; grade 1, mild gingivitis; grade 2, moderate gingivitis; grade 3, severe gingivitis; grade 4, gingivitis associated with proliferative and/or ulcerative lesions in the caudal oral cavity/palatoglossal fold and/or alveolar, labial, buccal, sublingual, and lingual mucosae (extra-gingival lesions). Only cats with grade 4 lesions were included in the FCGS group. Cats in the CH group presented with the absence of lesions or only mild gingivitis (six of 14 cats) at the time of examination.

### 4.2. Sample Collection, DNA Extraction, Library Preparation, and Sequencing

Samples were collected using a swab collection kit provided by MiDOG LLC, as previously described [44]. Briefly, oral samples from the FCGS cats were collected in mucosal transition areas (affected tissues and their contiguous normal areas) using a sterile DNA-free swab (HydraFlock^®^, Puritan^®^ Cat. No. 25-3406-H, Guilford, ME, USA). For the CH cats, swab samples were collected from the gingival, hard palate, rostral dorsal tongue, and other oral mucosal surfaces. All samples were immediately immersed and preserved in DNA/RNA Shield^TM^ (Zymo Research Corp.; Cat. No. R1108, Irvine, CA, USA) until processing at the MiDOG LLC testing facility (Irvine, CA, USA). Genomic DNA was purified using the ZymoBIOMICSTM-96 DNA kit (Cat. No. D4304, Zymo Research Corp., Irvine, CA, USA). Sample library preparation and data analysis for bacterial and fungal profiling were performed by Zymo Research Corp. using the Quick-16S NGS Library Prep Kit (Cat. No. D6400, Zymo Research Corp., Irvine, CA, USA), with minor modifications. Primer sequences are proprietary to the MiDOG LLC service and target the 16S rDNA V1–V3 region for bacteria and the ITS-2 region for fungal analysis. Libraries were sequenced using an Illumina HiSeq 1500 sequencer, and reads were filtered through Dada2 (R package version 3.4) [45]. Phylotypes were computed as percent proportions based on the total number of sequences in each sample. Relative abundances of bacteria compared to fungi were determined assuming an equivalency of one 16S rDNA copy to one fungal ITS copy. Species-level resolution of the sequencing approach used here has previously been demonstrated by shot-gun sequencing [44].

### 4.3. Statistical Analyses

Unless otherwise stated, results were expressed as median values with standard deviation. Measurements of alpha-diversity and evenness were calculated using the Shannon and Simpson indexes, and the number of observed species. Βeta-diversity was calculated using Bray–Curtis distance. Linear discriminant analysis (LDA) and effect size (LEfSe) were used to identify taxa that were significantly enriched in each group using the default settings [46]. Analyses of variance and false discovery rate control to correct for type I errors were performed using the default parameters of the program with *p* < 0.05 and the correlation coefficient (r) values of r > 0.60 (in absolute values) were considered significant (stats v3.6.1 R Core Team, R Foundation for Statistical Computing, Vienna, Austria, 2013). A presence-absence data matrix of species by site was generated by assuming species with abundance greater than 1% as presence and less than 1% as absent. The “coccur” function from R was used to generate pairwise classification of species having positive, negative, and random associations (“cooccur” package in R version 3.5.2, R Core Team, 2013).

## 5. Conclusions

Our data suggest that the mycobiota may have been inadvertently overlooked in the epidemiology of FCGS, and that feline oral health appears to be maintained by a complex equilibrium between bacterial and fungal populations that are part of the resident oral microbiota in cats. Once validated, NGS as a diagnostic tool is expected to be a more comprehensive, sensitive and faster technique than bacterial/fungal culture or PCR. Additional investigations need to be performed to further improve understanding of the oral ecosystem in cats and the vital biological interactions between bacteria and fungi in FCGS.

## Data Availability

Third Party Data Restrictions apply to the availability of these data. Data was obtained from MiDOG LLC and the data includes proprietary information regarding the sequencing specifics. Data for the abundance tables are available from the corresponding author (T Melgarejo) after written permission from MiDOG LLC has been granted, and upon reasonable request.

## Supplementary Materials

The following are available online at https://www.mdpi.com/article/10.3390/pathogens10070904/s1, Figure S1: LEfSe cladogram highlighting species that are significantly different between groups; Table S1: Average relative abundances of bacteria and fungi significantly different between FCGS and healthy feline samples.
