# Supplementary material for: Characterization of Oral Microbiota in Cats: Novel Insights on the Potential Role of Fungi in Feline Chronic Gingivostomatitis
Source: Pathogens. 2021 Jul 17;10(7):904. doi: 10.3390/pathogens10070904 (PMC8308807; doi:10.3390/pathogens10070904)
Supplement: Supplementary file 1 [file pathogens-10-00904-s001.zip › pathogens-1285870-supplementary.pdf]

Supplementary Table

Species	Oxygen dependence	FCGS		CH		LEfSe
		ReA (%)	Frequency	ReA (%)	Frequency	*P*-value
**Fungi**						
Saccharomyces cerevisiae	AE/MAE/AN	11.54	8	36.41	10	0.0307
Malassezia restricta	AE/MAE/AN	16.93	10	4.12	4	0.0001
Cladosporium penidielloides/salinae	AE/MAE/AN	8.48	4	0.00	0	0.0413
Malassezia arunalokei	AE/MAE/AN	6.78	7	1.31	2	0.0007
Aspergillaceae sp.	AE/MAE/AN	6.07	1	0.00	0	0.0275
**Bacteria**						
Bacteroidales sp. (order)	O-AN	5.97	9	2.57	14	0.0341
Xanthomonadaceae sp. (family)	AE	3.22	5	1.75	13	0.0079
Moraxella sp.	AE	3.18	9	8.46	12	0.0117
Arenimonas sp.	AE/F-AN	2.37	4	1.80	11	0.0177
Burkholderiaceae sp. (family)	AE/F-AN	2.01	8	1.59	13	0.0465
Alloprevotella sp.	O-AN	1.72	10	1.74	12	0.0007
Pseudoclavibacter sp.	AE	1.53	4	1.04	10	0.0392
Porphyromonas sp.	O-AN	1.29	13	2.76	14	0.0234
Fusibacter sp.	F-AN	1.11	10	0.98	14	0.0047
Lampropedia sp.	AE/F-AN	1.06	5	0.53	11	0.0470
Porphyromonas canoris	O-AN	0.89	8	2.36	13	0.0095
Pasteurellaceae sp. (family)	F-AN	0.72	8	1.77	13	0.0041
Fusobacterium sp.	(O)-AN	0.69	11	1.19	14	0.0047
Burkholderiales sp. (order)	AE/F-AN	0.55	10	2.14	14	0.0015
Bergeyella zoohelcum	AE	0.36	4	2.80	12	0.0002
Bacteroides sp.	O-AN	0.28	11	1.54	13	0.0128
Flavobacterium sp.	AE	0.20	1	1.01	10	0.0008
Flavobacteriaceae sp. (family)	AE	0.16	2	2.39	12	0.0001
Peptostreptococcus canis	O-AN.	6.87	10	2.27	4	0.0080
Fretibacterium sp.	O/F-AN	3.73	13	1.10	14	0.0069
Propionibacteriaceae sp. (family)	AE/F-AN	3.71	10	0.66	14	0.0346
Spirochaetaceae sp. (family)	O/F-AN	2.27	12	1.29	13	0.0416
FamilyXIII sp. (family)	O-AN	1.79	9	0.58	4	0.0074
Fusobacterium nucleatum	O-AN	1.30	8	0.60	3	0.0420
Lachnospiraceae sp. (family)	O-AN	1.11	11	0.36	13	0.0476
Methanobrevibacter oralis	O-AN	1.07	6	1.87	1	0.0462

Abbreviations; AE: Aerobic; MAE: microaerobic; AN.: Anaerobic; F: Facultative; O: Obligate; ReA: Relative Abundance

Table S1. Average relative abundances of bacteria and fungi significantly different between FCGS and CH feline samples. Frequency lists how many samples in each group had that given species present. Those with a grey background were significantly enriched in FCGS samples. For brevity of the table, only those species that had at least 1% relative abundances in one the two groups are shown.

Supplementary Figure

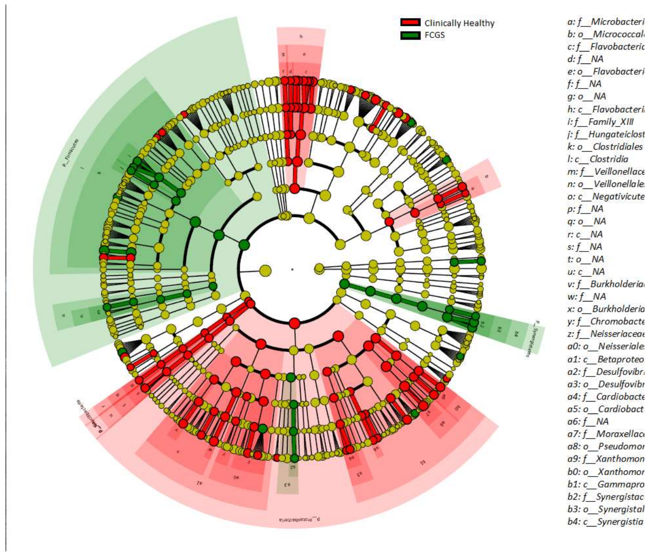

(**a**)

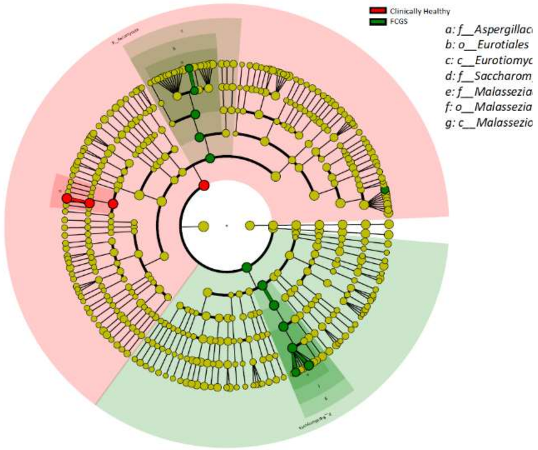

(**b**)

Figure S1. LEfSe cladogram highlighting species that are significantly different between groups for (a) bacteria and (b) fungi. Each ring represents a taxonomic level, with the phylum being in the center and species on the outermost ring. Nods colored in moss green are not significantly different between groups. The large number of identified taxa limits the readability of the graph. Therefore, those species that represented on average at least 1% of the microbiota per group are listed in Table S1. Taxa significantly enriched in FCGS samples are shown in green, taxa significantly enriched in CH samples are shown in red.
